# *In vivo* validation of an ultra-high field, high temporal resolution myocardial tagging technique for assessment of diastolic function in mice

**DOI:** 10.1186/1532-429X-15-S1-P129

**Published:** 2013-01-30

**Authors:** Jaehoon Chung, Hong Liu, Euy-Myoung Jeong, Lianzhi Gu, Scott Gladstein, Afshin Farzaneh-Far, E Douglas Lewandowski, Samuel Dudley

**Affiliations:** 1 University of Illinois at Chicago, Chicago, IL, USA

## Background

Heart failure with preserved ejection fraction accounts for approximately half of all heart failure cases and is associated with similar morbidity and mortality. Although abnormalities of diastolic function are felt to play an important role, no specific treatments have been identified for this common condition, primarily because of poor understanding of its pathophysiology. Despite development of several murine models of this disease, accurate non-invasive assessment of diastolic function has been challenging. Echocardiographic measurements have been limited by small heart sizes, rapid ventricular rates and high inter-observer variability. The aim of this study was to assess the ability of ultra-high field, high temporal resolution CMR tagging to assess diastolic function in mice, compared to the gold-standard technique of invasive pressure-volume loop analysis.

## Methods

**Figure 1:**
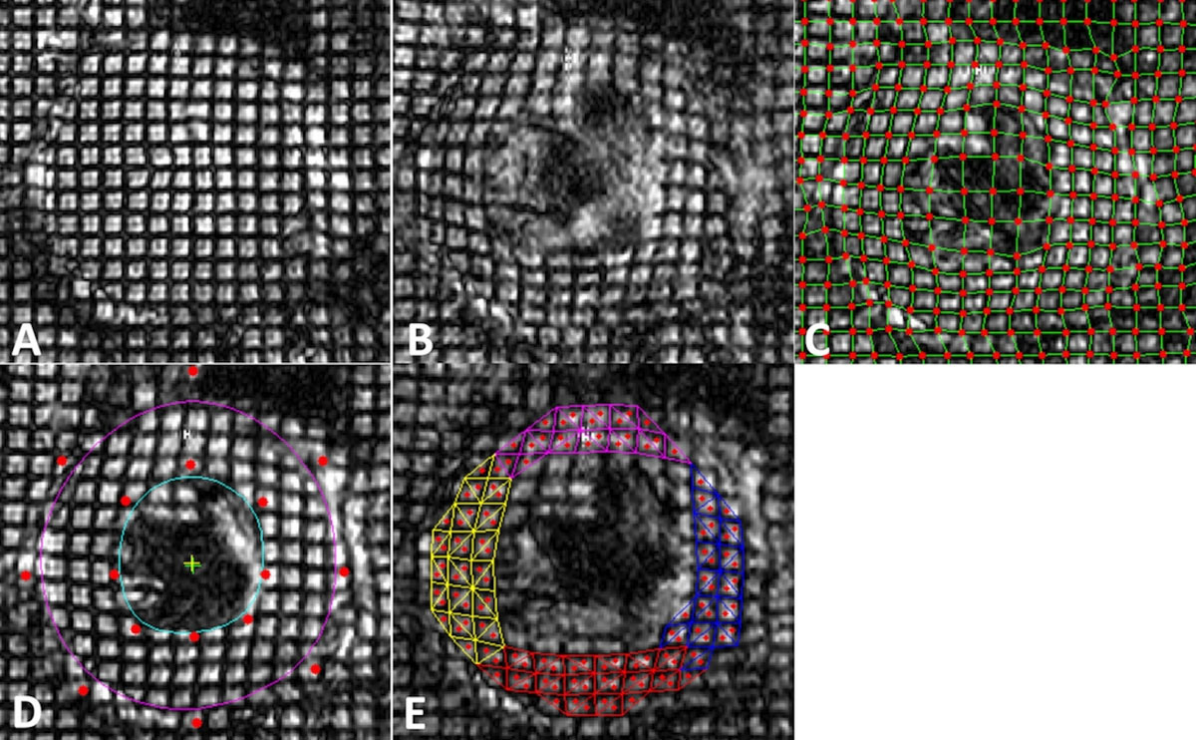
Homogeneous strain analysis of myocardial tagged images. Cardiac and respiratory gated SPAMM-tagged images were obtained at the mid-ventricular short axis from end-systole (A) to end-diastole (B) with the following parameters: FOV 20 mm, slice thickness 1 mm, echo time 1.5 ms, temporal resolution 5 ms, flip angle 30 degrees, acquisition matrix 256x256, number of excitations=4. Each tagged image was overlaid with manually traced tagged lines and intersections (C). Endo- and epicardium was contoured using the B spline method to define the direction of circumferential and radial strain at 8 different points as indicated by red dots (D). Triangulation of myocardium allowed diastolic homogeneous strain analysis and calculation of circumferential strain (Ecc) rate (E).

High fat diet-induced diabetic mice (n=7) and age-matched control mice (n=4) underwent tissue tagged CMR using a 14.1-Tesla, 89 mm vertical bore magnet with a 600 MHz birdcage resonator. Myocardial tagged images were obtained at the mid-ventricular short axis from end-systole to end-diastole using a spatial modulation of magnetization (SPAMM) sequence (Figure 1A and B). Tagged images were processed using a Matlab-based program to calculate maximal circumferential strain (Ecc) rate (Figure 1C, D and E). Concurrent invasive pressure-volume loop analysis was performed within 24 hours using 1.4 Fr pressure-volume catheters. The end-diastolic pressure volume relationship (EDPVR) was calculated by acquiring pressure-volume loops following acute compression of the inferior vena cava.

## Results

**Figure 2:**
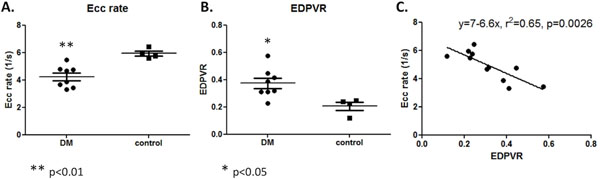
A) CMR measured circumferential strain (Ecc) rate is significantly reduced in diabetic mice (4.24 ± 0.28 1/s) compared with controls (5.94 ± 0.19 1/s). B) End-diastolic pressure volume relationship (EDPVR) measured by invasive pressure-volume loop analysis shows a significantly steeper EDPVR in diabetic mice (0.38 ± 0.04) compared with controls (0.21 ± 0.03). C) Relationship of invasive EDPVR to CMR measured Ecc rate.

LV ejection fraction was similar in both groups (65.8 ± 1.8 % vs. 65.3 ± 1.6 %, p=0.86). However, Ecc rate was significantly reduced in diabetic mice compared with controls (4.24 ± 0.28 1/s vs. 5.94 ± 0.19 1/s, p<0.01), indicating significant relaxation impairment during the rapid filling phase in diabetic mice (Figure 2A). Pressure-volume loop analysis showed that diabetic mice had a significantly steeper EDPVR compared with controls (0.38 ± 0.04 vs. 0.21 ± 0.03, p<0.05), confirming diastolic dysfunction in the diabetic mice (Figure 2B). There was a significant correlation between Ecc rate and EDPVR (r2 = 0.65, p<0.01, Figure 2C).

## Conclusions

This study provides preliminary validation of ultra-high field, high temporal resolution CMR tagging for non-invasive assessment of diastolic function in mice compared to the gold standard of invasive pressure-volume loop analysis. This technique has potential for assessing the effects of putative therapeutic compounds on mice models of diastolic dysfunction.

## Funding

This work was supported by NIH R01 and T32.

